# The effect of metformin on influenza vaccine responses in nondiabetic older adults: a pilot trial

**DOI:** 10.1186/s12979-023-00343-x

**Published:** 2023-05-02

**Authors:** Dominique E. Martin, Andreia N. Cadar, Hunter Panier, Blake L. Torrance, George A. Kuchel, Jenna M. Bartley

**Affiliations:** 1 UConn Center On Aging, University of Connecticut School of Medicine, 263 Farmington Avenue, Farmington, CT 06030, 860-679-8322 USA; 2 Department of Immunology, University of Connecticut School of Medicine, 263 Farmington Avenue, Farmington, CT 06030, 860-679-8322 USA; 3 Department of Medicine, University of Connecticut School of Medicine, Farmington Avenue, Farmington, CT 06030 USA

**Keywords:** Metformin, Aging, Vaccination, Immune exhaustion, Geroscience-guided clinical trial

## Abstract

**Background:**

Aging is associated with progressive declines in immune responses leading to increased risk of severe infection and diminished vaccination responses. Influenza (flu) is a leading killer of older adults despite availability of seasonal vaccines. Geroscience-guided interventions targeting biological aging could offer transformational approaches to reverse broad declines in immune responses with aging. Here, we evaluated effects of metformin, an FDA approved diabetes drug and candidate anti-aging drug, on flu vaccination responses and markers of immunological resilience in a pilot and feasibility double-blinded placebo-controlled study.

**Results:**

Healthy older adults (non-diabetic/non-prediabetic, age: 74.4 ± 1.7 years) were randomized to metformin (*n* = 8, 1500 mg extended release/daily) or placebo (*n* = 7) treatment for 20 weeks and were vaccinated with high-dose flu vaccine after 10 weeks of treatment. Peripheral blood mononuclear cells (PBMCs), serum, and plasma were collected prior to treatment, immediately prior to vaccination, and 1, 5, and 10 weeks post vaccination. Increased serum antibody titers were observed post vaccination with no significant differences between groups. Metformin treatment led to trending increases in circulating T follicular helper cells post-vaccination. Furthermore, 20 weeks of metformin treatment reduced expression of exhaustion marker CD57 in circulating CD4 T cells.

**Conclusions:**

Pre-vaccination metformin treatment improved some components of flu vaccine responses and reduced some markers of T cell exhaustion without serious adverse events in nondiabetic older adults. Thus, our findings highlight the potential utility of metformin to improve flu vaccine responses and reduce age-related immune exhaustion in older adults, providing improved immunological resilience in nondiabetic older adults.

**Supplementary Information:**

The online version contains supplementary material available at 10.1186/s12979-023-00343-x.

## Background

Aging is a major risk factor for most common chronic diseases, and older adults, especially those with multiple chronic conditions, are at the greatest risk of hospitalization, disability and death following influenza (flu) infection [1]. In fact, almost 90% of flu-related deaths in the United States occur in people over 65 years old [2, 3]. Vaccination is the most effective way to prevent infectious disease and/or reduce severity of infectious disease illness. However, age-related immune dysregulation is known to reduce vaccine efficacy, leaving older adults less protected in terms of severe infection compared to younger populations [4, 5]. Diminished protection in older adults is due to both inefficient cell-mediated and humoral immune responses evident through lower antibody titers and reduced T cell proliferation [6, 7]. Together, these factors impact the level of immunological protection generated in response to vaccination and with age. Despite year-to-year variability in efficacy, flu vaccine induced protection is consistently reduced in older adults compared with younger populations [8]. Fortunately, specific flu vaccines have been developed to overcome these vaccination deficits in older adults. Currently, two vaccines are FDA approved for adults 65 years of age and older in the United States including a high-dose vaccine and adjuvanted vaccine. The high-dose flu vaccine contains four times the antigen dose compared with the standard dose which significantly increases antibody titers, seroconversion rates, and importantly, better protects against hospitalization and death in older adults [9, 10]. Additionally, an adjuvanted flu vaccine that includes MF59 also leads to significantly higher antibody production and enhances overall protection in older adults [11, 12]. While these vaccines provide improved protection compared to the standard dose flu vaccine in older adults [13], the burden of flu remains high, and overall flu vaccine efficacy is still inadequate in this population.

Recently, geroscience-focused research has highlighted that targeting of biological aging may be a more successful approach to alleviate multiple aspects of age-related declines in physiology and resilience in the face of common stressors, thus improving overall healthspan. While targeting specific deficits in the flu vaccine response has been fruitful, the response to vaccination is highly coordinated, and diminished responses in older adults are likely not due to a singular deficit, but rather a multitude of age-related declines. Thus, targeting the hallmarks of aging [1] may be a more suitable approach to improve vaccination responses. Two of the hallmarks of aging, mitochondrial dysfunction and dysregulated nutrient sensing, impact metabolic function, which changes with aging at both the systemic and cellular level [14-16]. Mammalian target of rapamycin (mTOR) and AMP-activated protein kinase (AMPK) are considered master regulators of metabolism and are actively being studied in relation to immune responses, which has quickly developed into its own field, termed immunometabolism. Indeed, proper immune cell function is dependent on proper cellular metabolism. For instance, modulation of the mTOR/AMPK pathways is essential for T cell activation, T helper (Th) subset differentiation, effector function, and memory cell generation [17-19]. Importantly, recent research has highlighted age-related changes in immune cell metabolism [20, 21] with CD4 T cells from older adults having higher baseline and maximal oxygen consumption rate compared to young CD4 T cells [20]. Thus, we postulated that targeting aging biology by combating metabolic dysregulation and altered nutrient sensing will lead to overall improvements in the immune responses of older adults, ultimately improving vaccine responses and immunological resilience.

Drugs that target metabolism by modulating AMPK/mTOR pathways, including metformin and rapamycin, have been of keen interest in combating various age-related diseases. Metformin is an FDA approved diabetes drug that modulates these pathways and other metabolic targets. Importantly, metformin targets almost all aspects of the hallmarks of aging, making it an ideal candidate to target the biology of aging overall [22]. In terms of improving immune responses, metformin has shown positive effects on various immune cell types both through direct immunometabolic modulation, as well as through the alleviation of chronic pro-inflammatory signaling known to dysregulate immune responses [23]. In young mice, metformin increases CD8 T cell memory formation through AMPK activation and fatty acid oxidation enhancement [24, 25]. In aged human CD4 T cells, metformin improves mitochondrial function, enhances autophagy, and alleviates age-related Th17 driven inflammation [20]. Metformin treatment enhanced in vitro and in vivo B cell responses in diabetics, evidenced by improved antibody responses, compared with diabetics on other hypoglycemics [26]. It was also shown that in older adults with type II diabetes, metformin enhanced B cell function and antibody responses to flu vaccination specifically [27]. Contrarily, one study showed impaired flu vaccine responses in older adults on chronic metformin treatment compared with those not on any long term medication. However, this study had a small sample size of older diabetic adults on metformin (*n* = 5), examined limited time points post vaccination, and did not provide details on additional comorbidities and group differences, making results difficult to interpret [28]. Nevertheless, in the context of many diseases, metformin was shown to reduce the expression of pro-inflammatory cytokine/chemokine genes [29-31]. Thus, in many cases, metformin was shown to improve overall immune responses and reduce inflammation at least partially by promoting the formation of M2 macrophages and T regulatory cells [32]. These various studies support metformin as a potential candidate to improve immunological resilience and control inflammation with aging [23].

Furthermore, a similar approach that targeted aging and metabolism concurrently in older adults was previously successful in improving immune responses. Healthy older adults who received a rapamycin analogue had increased flu vaccine antibody responses and overall immune responses compared to those who received placebo [33, 34]. Interestingly, the rapamycin analogue was discontinued 2 weeks prior to flu vaccination [35] potentially due to concerns of acute immune suppression that has been previously shown with high doses of rapamycin [36]. Therefore, metformin’s well-documented safety profile and minimal side effects noted over decades of use in diabetics may be a more optimal intervention to improve immune responses of older adults. Multiple lines of research support the ability of metformin to improve metabolism [37] and age-related dysfunction [22, 23, 38]. However, to date, the effect of metformin on immune responses to flu vaccination in healthy older adults who are not diabetic or prediabetic has not been studied.

While serum antibody titers are considered the gold standard to assess overall flu vaccine responses, other approaches can provide additional information on cellular responses. Seminal work from the lab of Dr. Janet McElhaney’s has shown that T cell responses to flu vaccination may be more indicative of immunological protection against infection in older adults [39-41]. Indeed, ex vivo stimulation of peripheral blood mononuclear cells (PBMCs) with live flu virus allows evaluation of Granzyme B (GrB) induction and cytokine secretion, specifically IFN-γ and IL-10, to assess cell-mediated responses [39, 40] and flu vaccine induced protection. Similarly, circulating PBMCs provide insight into cell activation and differentiation patterns in response to flu vaccination. While vaccine responses occur within the secondary lymphoid tissues, certain circulating immune cells have been shown to be related to responses occurring within the lymphoid tissue. Circulating T follicular helper cells (cTfh, CD4 + CXCR5 +), similar to Tfh within the lymphoid tissue, can support antibody secretion [42]. With aging, cTfh from older adults have reduced frequency, decreased in vitro B cell help ability, and increased expression of inducible T cell costimulatory (ICOS) at baseline compared with young adults [43]. Importantly, in young adults, ICOS expression on cTfh increases in response to flu vaccination and corresponds to flu IgM and IgG responses. Conversely, older adults fail to upregulate ICOS on cTfh and this is not correlated with antibody responses, suggesting a failure in cTfh to respond to flu vaccination in older adults [43]. Thus, to thoroughly assess how age and/or therapies can impact flu vaccination responses, it is necessary to have a comprehensive approach that considers multiple aspects of vaccine responses rather than relying on antibody titers alone.

Targeting the physiology of aging and metabolism with metformin may be a novel way to improve systemic inflammation, immune cell exhaustion, and overall immune responses resulting in enhanced flu vaccine induced protection in older adults. To further understand these relationships, we designed a double-blinded placebo-controlled pilot study to determine the effect of metformin on flu vaccine responses in healthy (nondiabetic and nonprediabetic) older adults. Here, we show that metformin had limited side effects and improved some key aspects of flu vaccine responses and overall immune exhaustion in healthy older adults. Thus, metformin may represent a novel way to improve vaccine responses and reduce risk to flu infection and associated burden in older adults.

## Results

### Subject characteristics and treatment tolerability

**Fig. 1:**
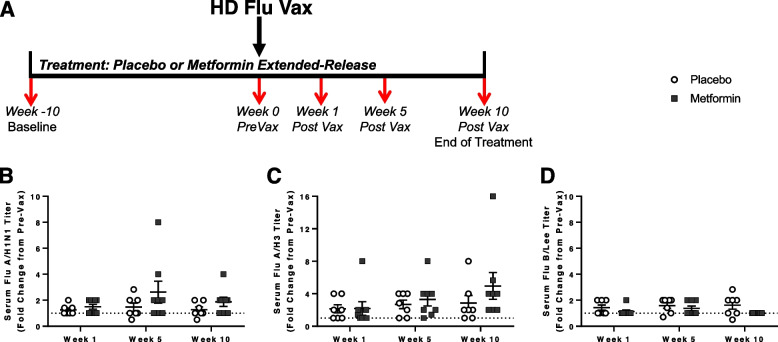
Experimental design and assessment of serum antibody titers. **A**) Experimental design. Patients were randomized to metformin (final dose 1500 mg ER/day) or placebo and started with 1 tablet/day for week 1 (500 mg metformin ER/day or placebo), then 2 tablets a day for week 2 (1000 mg metformin ER or placebo), and finally 3 tablets a day for week 3 (1500 mg metformin ER or placebo) until the completion of the study. Participants were vaccinated with high-dose flu vaccine after ~ 10 weeks of treatment. Blood was drawn prior to treatment (week -10, Baseline), immediately prior to vaccination (week 0), and 1-, 5-, and 10-weeks post vaccination. Serum was analyzed via hemagglutinin inhibition assay for flu antibody titers. Geometric Mean Titers (GMT) were normalized to pre-vaccination (Pre-Vax) levels to calculate fold change for the **B**) A/Kansas/14/2017 (H3N2)-like virus, **C**) A/Brisbane/02/2018 (H1N1)pdm09-like virus and **D**) B/Colorado/06/2017-like virus (B/Victoria lineage) contained in the seasonal flu vaccine. The dashed line denotes a fold change of 1. Statistical significance was calculated by two-way repeated measures ANOVA with Šídák’s posthoc corrections and significance set at *p* < 0.05

Healthy nondiabetic and nonprediabetic older adults were randomized to metformin extended release (ER) or placebo for ~20 weeks and were vaccinated with high-dose flu vaccine following ~10 weeks of treatment (Fig. 1A, details in **Methods**). Baseline characteristics between the treatment arms were similar (Table 1). Overall, patients were relatively healthy with no differences in age or gender distribution between groups. Of note, participants in both treatment groups had limited co-morbidities and were considered non-frail by both the Rockwood Frailty Index [44] and Fried Frailty Phenotype Frailty index [45]. Participants also were considered robust from the validated short performance physical battery (SPPB) [46]. There were no differences in height, weight, or BMI at baseline or following treatment between treatment groups.

**Table 1:** Participant demographics and characteristics at baseline and following 20 weeks of treatment

	Placebo	Metformin
Subjects	7	8
Age	74.71 ± 2.45	74.13 ± 2.42
Male	3, 43%	5, 63%
Number Comorbidities Per Subject (#)	2.57 ± 0.84	2.50 ± 0.65
Height (inches)	64.43 ± 2.09	66.73 ± 1.13
Baseline Weight (lbs)	159.43 ± 7.52	167.86 ± 12.23
End of Treatment Weight (lbs)	162.71 ± 8.24	164.18 ± 13.09
Baseline BMI (kg/m^2^)	27.31 ± 1.68	26.43 ± 1.47
End of Treatment BMI (kg/m^2^)	27.83 ± 1.74	25.80 ± 1.56
Baseline Rockwood Frailty Index	0.09 ± 0.007	0.08 ± 0.003
End of Treatment Rockwood Frailty Index	0.09 ± .005	0.09 ± 0.005
Baseline Fried Frailty Score	0.86 ± 0.26	0.75 ± 0.25
End of Treatment Fried Frailty Score	1.29 ± 0.29	0.75 ± 0.16
Baseline SPPB Score	11 ± 0.69	10.63 ± 0.71
End of Treatment SPPB Score	11.43 ± 0.30	11.43 ± 0.28

Participants were treated with placebo or metformin for approximately 20 weeks. Frailty index was calculated via 40 items previously validated for influenza outcomes [44]. Fried Frailty score was calculated via standard 5 categories [45]. The validated short performance physical battery (SPPB) was also utilized [46]. Data presented as mean ± SEM and analyzed via t-test. No significant differences were observed between groups

In general, metformin treatment was well tolerated by study participants. No deaths or serious adverse events (AEs) occurred during the study. AEs experienced by each treatment group are detailed in Supplementary Table 1 with no clear patterns attributed to metformin and relatively equal occurrence in metformin and placebo groups. Clinical safety labs did not reveal any major changes with metformin or placebo treatment.

### Flu vaccine led to increased antibody titers with no differences observed between groups

Serum flu antibody titers were analyzed via hemagglutination inhibition assays (HIA). HIA titers assess the functional antibody activity and are considered the gold-standard marker as a correlate of protection against flu illness [47-49]. HIA antibody fold changes were calculated from pre-vaccination levels (Fig. 1B-D). Induction of flu antibodies post vaccination was greater for the H3N2 strain (~ threefold increase from pre-vax levels) compared with H1N1 and B flu strains. Flu B strain had minimal induction (only ~ 1.3 fold increase from pre-vax levels) as others have previously noted [50, 51]. No significant differences between treatments were observed, however it is important to note that our study was not powered to detect differences in antibody titers due to the low sensitivity of the HIA assay and high level of variability in responses, especially in older adults [52]. Thus, future research is necessary to assess the effect of metformin on flu antibody titers, as well as antibody quantity and quality, in healthy older adults.

### Flu vaccine induced cell-mediated responses in both metformin and placebo participants

**Fig. 2:**
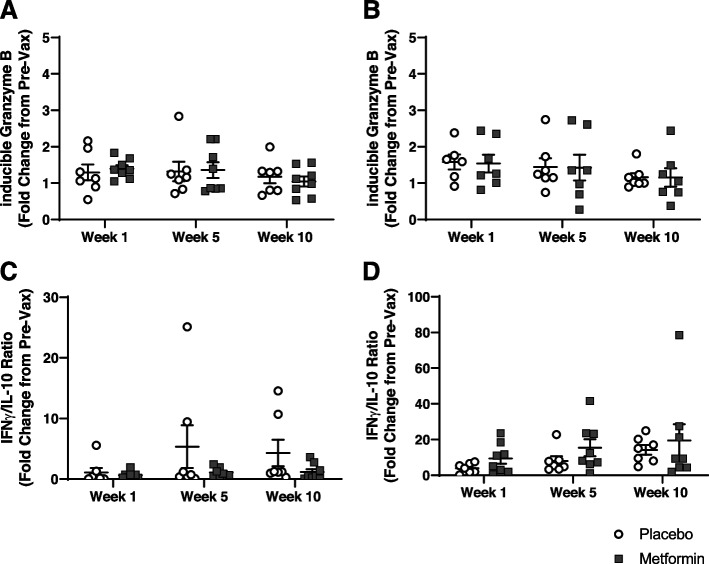
Assessment of ex vivo cell-mediated vaccination responses. PBMCs were stimulated with live flu virus and cell-mediated responses were analyzed in the supernatants and lysates. Inducible Granzyme B (iGrB) levels were calculated in lysates by normalizing to total protein content and unstimulated normalized GrB levels. iGrB and IFNy/IL-10 ratio are presented as fold change at given week post vaccination compared to pre-vaccination levels. No differences were observed in iGrB in response to influenza **A**) AVic/H3N2 or **B**) BLee strain or in IFNy/IL-10 responses to **C**) Avic/H3N2 or **D**) Blee strains. Statistical significance was calculated by two-way repeated measures ANOVA with Šídák’s posthoc corrections and significance set at *p *< 0.05

Cell mediated immune response have previously been shown to be correlated with protection against flu infection in older adults [40, 49, 53]. PBMCs were stimulated with live flu virus and iGrB in the lysates were calculated as fold changes from pre-vaccination levels. No differences in iGrB concentrations were observed between treatment arms (Fig. 2A and 2B). Additionally, IFNy/IL-10 ratios were measured in the supernatant and fold changes were calculated from pre-vaccination level. No differences in IFNy/IL-10 ratios were observed between treatment arms (Fig. 2C and 2D). While clear patterns of flu vaccine responses were observed with cell-mediated responses, no differences were seen between treatments.

### Metformin treatment enhanced circulating Tfh responses to flu vaccine

**Fig. 3:**
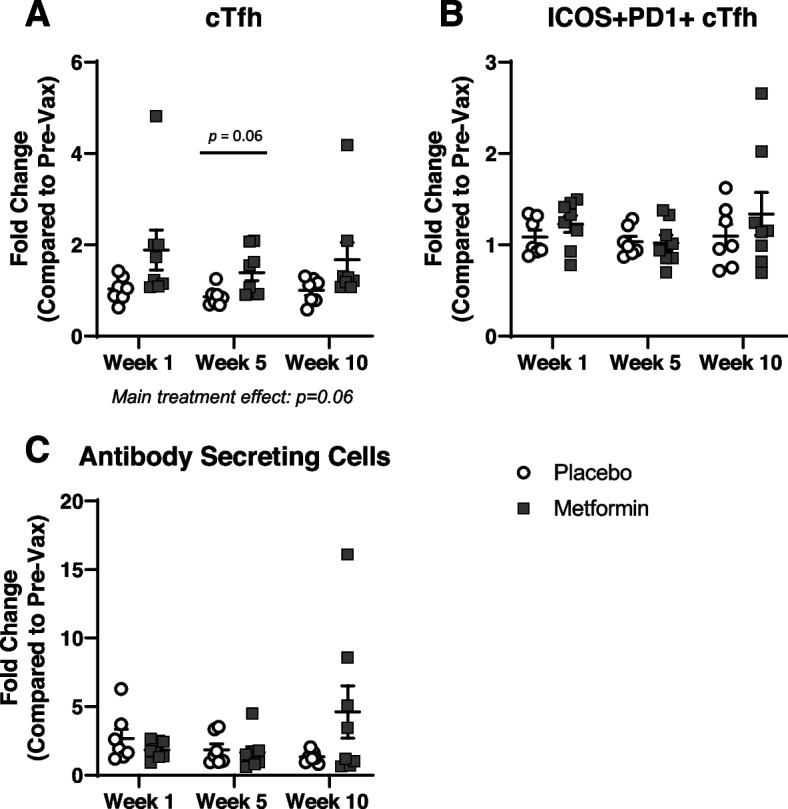
Flow cytometry analysis of circulating PBMC responses to flu vaccination. Peripheral blood mononuclear cells (PBMCs) were analyzed for **A**) circulating T follicular helper cells (cTfh), **B**) activated ICOS + PD1 + cTfh, and **C**) Antibody Secreting Cells (ASCs). Data are presented as fold change at given week post vaccination compared to pre-vaccination levels. A trending main metformin treatment effect was observed in cTfh. Statistical significance was calculated by two-way repeated measures ANOVA with Šídák’s posthoc corrections and significance set at *p* < 0.05

To comprehensively assess flu vaccine responses, we analyzed circulating Tfh and antibody secreting cells (ASCs). Fold change in frequency of cell populations following vaccination was calculated from pre-vaccination levels. cTfh, which have been shown to support B cell antibody secretion [42], were identified as CD4 + CXCR5 + . Metformin had a trending treatment effect (*p* = 0.06) of greater fold increase in cTfh following vaccination compared to placebo. Moreover, there was also a trending (*p* = 0.06) increase in the fold change of cTfh at 5 weeks post vaccination compared to placebo (Fig. 3A). Activation status of cTfh was assessed via ICOS and programmed death receptor 1 (PD-1) expression, however, were not different between groups (Fig. 3B). Similarly, there were no differences in mean fluorescence intensity (MFI) of ICOS or PD-1 on cTfh (Supplemental Fig. 1). The role of increased cTfh without increased activation is unclear. Future research with a larger sample size is necessary to determine how increased cTfh can impact vaccination responses. Fold change in circulating ASCs (identified as CD19 + CD38^Hi^CD24- [54]) were increased following vaccination in both treatment groups, however no differences existed between groups (Fig. 3C). Thus, metformin treatment showed a strong trend to improve cTfh vaccination responses, however no significant differences were observed in antibody responses with treatment. Additionally, we analyzed raw frequency of these populations and did not see any differences in cTfh, cTfh activation, or ASCs (Supplemental Fig. 2A-C). Further, when we examined fold changes of these cell populations from baseline, we did not observe any significant impact of metformin on cTfh or ASCs prior to vaccination (Supplemental Fig. 2D-F). Moreover, we did not observe any differences in naïve, central memory, effector memory, or terminally differentiated effector memory cells re-expressing CD45RA (TEMRA) CD4 or CD8 T cells populations (Supplemental Fig. 3A-H) or in naïve, switched memory B cells, non-switched memory B cells, or double negative B cells (Supplemental Fig 3I-L) with metformin treatment prior to vaccination. This suggests that there is an interaction between vaccination and metformin treatment, and that the differences we observe are due to metformin’s impact on vaccination responses, rather than impact on baseline circulating cell populations. Notably, no negative effects were evident with metformin in terms of flu vaccine responses or adverse events. Due to the large variability in flu vaccine responses between individuals and increased heterogeneity in older populations, a larger sample size is necessary to determine if metformin can improve overall flu vaccine responses in healthy older adults.

### Metformin reversed some aspects of age-related CD4 T cell exhaustion, circulating markers of inflammation, and biomarkers of aging

**Fig. 4:**
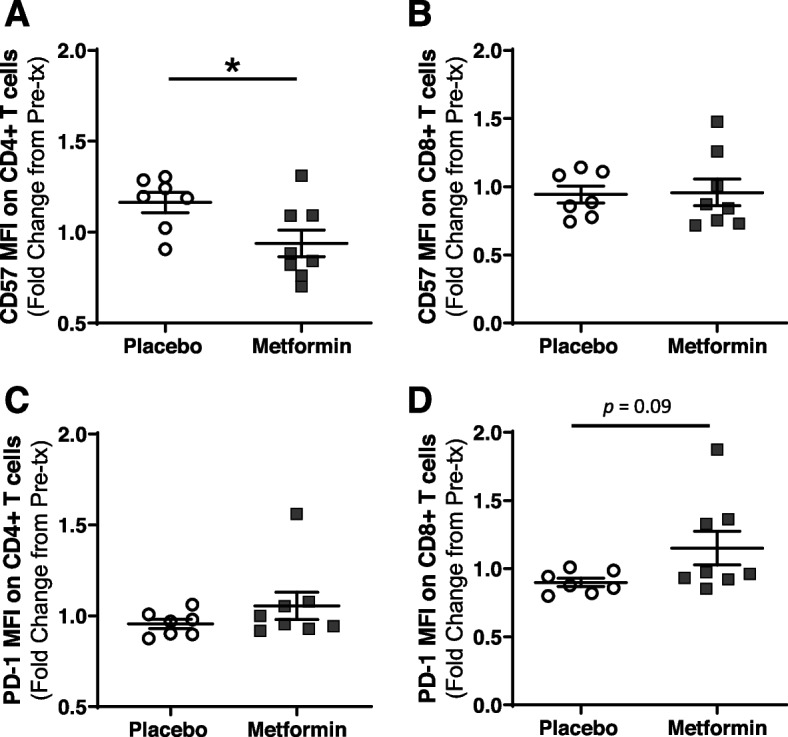
Flow cytometry analysis of exhaustion markers. CD4 and CD8 T cells were analyzed for expression of exhaustion markers CD57 and PD-1 prior to and following 20 weeks of treatment with either placebo or metformin and fold change in mean fluorescent intensity (MFI) was calculated. CD4 T cells had reduced **A**) CD57 expression with metformin, while no differences in **C**) PD-1 expression were observed. CD8 T cells had no differences in **B**) CD57 expression, while there was a trending increased in **D**) PD-1 expression with metformin treatment. Statistical significance was calculated by t-test and significance set at *p* < 0.05

Thus, we next sought to determine if 20 weeks of metformin treatment had an effect on markers of T cell exhaustion and circulating inflammatory markers. We evaluated the fold change in PD-1 and CD57 MFI on CD4 and CD8 T cells from pre-treatment levels. Both PD-1 and CD57 have been previously shown to indicate T cell dysfunction and exhaustion [55, 56]. PD-1 has been shown to be a marker of exhausted immune cells, particularly T cells, with implications in both chronic disease and acute infection [57, 58]. Moreover, both CD57 and PD-1 expression have been shown to increase on the T cells of older adults and are implicated as major drivers of dysregulated responses and immunosenescence [59]. In our study, patients treated with metformin had reduced CD57 MFI expression on CD4 T cells when compared to placebo (Fig. 4A). In contrast, no difference in CD57 expression was noted on CD8 T cells (Fig. 4B) and no differences in frequency of CD57 + CD4 or CD8 T cells were observed (Supplemental Fig. 4). CD57 has been used to identify T cells that are terminally differentiated and have low proliferative capacity [55, 56], thus metformin treatment reduced one aspect of immunosenescence in CD4 T cells. In contrast, unlike CD57, metformin did not decrease another exhaustion marker PD1 in CD4 T cells. Conversely, we observed a non-significant increase in PD1 on CD8 T cells (*p* = 0.09, Fig. 4D). More research is necessary to elucidate the differential effects of metformin on these two markers of exhaustion in CD4 T cell as opposed to CD8 T cells and overall immunosenescence.

**Fig. 5:**
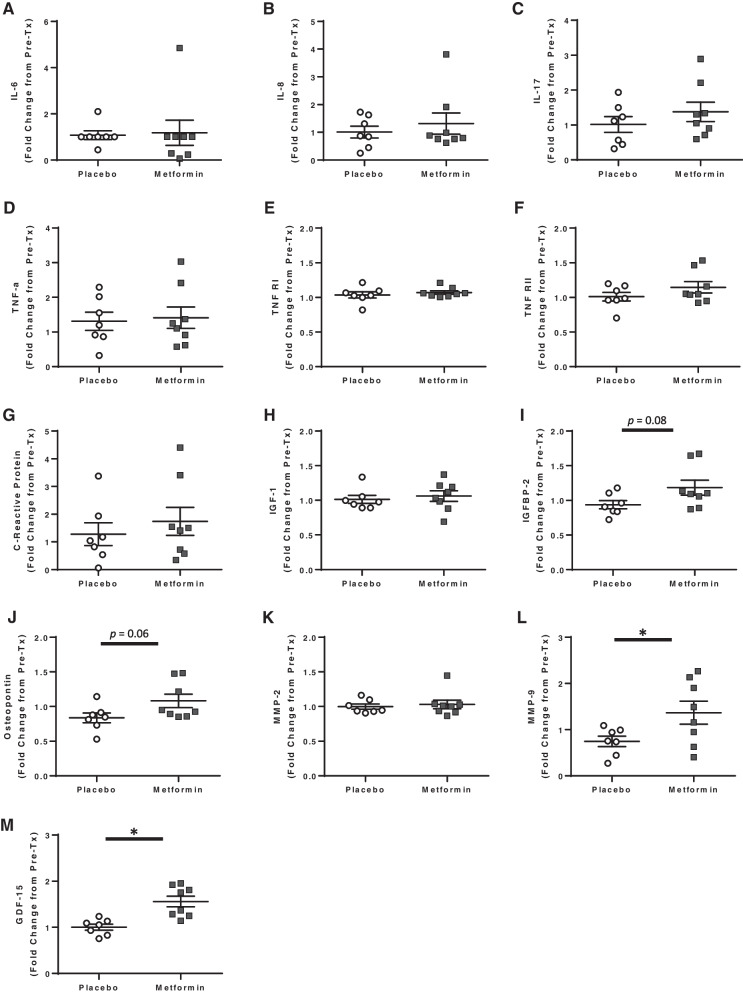
Analysis of circulating markers of inflammation and aging. Circulating markers of inflammation, growth factors, and age-related factors were measured prior to and following 20 weeks of treatment with either placebo or metformin. Plasma **A**) IL-6, **B**) IL-8, **C**) IL-17A, **D**) TNFa, **E**) TNFRI, **F**) TNFRII, **G**) C-reactive protein, **H**) IGF-1, **I**) IGFBP-2, **J**) Osteopontin, and **M**) GDF-15 and serum **K**) MMP-2 and **L**) MMP-9 was measured and fold change was calculated. Statistical significance was calculated by t-test and significance set at *p* < 0.05

While many studies have shown that metformin decreases a variety of inflammatory factors in diabetics [29, 30, 60], the utility of metformin to reduce age-related inflammation in healthy older adults has not been previously explored. Thus, we evaluated changes in circulating inflammatory markers from baseline and after 20 weeks of treatment. In this study, metformin did not alter inflammation markers IL-6, IL-8, IL-17A, TNFα, TNFRI, TNF RII, or CRP (Fig. 5A-G), which have previously been noted as markers of age-associated inflammation [61-63]. Additionally, metformin treatment has been shown to reduce insulin-like growth factor-1 (IGF-1) signaling in certain scenarios [22, 64], and reduced IGF-1 has been associated with increased longevity and healthspan in multiple animal models [22, 65]. While no differences were observed in circulating IGF-1 levels (Fig. 5H), metformin treatment trended to increase IGF binding protein (BP)-2 (Fig. 5I), similar to previous observations in diabetics [66]. We also evaluated other circulating factors known to be affected by metformin use in diabetics and related to aging/longevity [29, 64, 66-70]. Metformin treatment increased osteopontin, GDF-15, and MMP9 compared with placebo, but had no effect on MMP2 levels (Fig. 5J-M). GDF15 has been shown to mediate the effects of metformin on body weight and energy balance [67]. Metformin has previously been shown to reduce MMP2 and MMP9 in certain in vitro studies [69, 71] and diabetics on metformin have decreased serum MMP-9 [72]. Thus, it seems that the effects of metformin on these circulating factors may be context specific.

## Discussion

The primary goal of this study was to determine the feasibility, safety, and utility of metformin as a tool to improve immune responses to vaccination in healthy nondiabetic, nonprediabetic older adults. Importantly, metformin was well-tolerated among participants and did not have any serious adverse events during this study, demonstrating that the off-label use of metformin in this cohort was safe and feasible.

Increased flu antibodies were observed in both the metformin and placebo treatment groups following vaccination. Since our study was not powered to discern differences in antibody responses, no statistical differences were noted between treatment groups. Previous studies have found that metformin can enhance B cell function and antibody responses of older adults with type 2 diabetes mellitus [27]. However, it is unclear if these improvements are evident in nondiabetic and nonprediabetic older adults. It is possible that metformin may only improve diabetic B cell deficits, but not age-related B cell deficits in a generally healthy, non-diabetic, older population. Additional studies with larger sample sizes (> 100 subjects/group) are necessary to evaluate the effect of metformin on flu vaccine antibody responses. Further, while HIA titers are considered the gold-standard marker as a correlate of protection against flu illness [47-49], they quantify functional antibody against the flu hemagglutinin (HA) and not the entire repertoire of antibody responses and overall quality of antibodies. Additional measures of antibody quantity and quality, including more cutting edge OMIC strategies [73], will be particularly useful to shed light on to how metformin may modulate humoral responses to flu vaccination.

It is important to note that our subject population was exceptionally healthy and may not be reflective of the general aged population, as they had limited co-morbidities and low frailty scores (Table 1). Therefore, it is possible that the effects of metformin on B cell and antibody responses may be more evident in populations with more co-morbidities, and therefore a greater contribution of biological aging to their altered immune function, such as those who are pre-frail, frail, or prediabetic. Interestingly, approximately 23% of older adults screened had undiagnosed prediabetes, indicative of the high burden of prediabetes among otherwise healthy older adults. While we intentionally excluded those with prediabetes to reduce the confounding variable of metformin’s glucose lowering ability, it is possible we unintentionally excluded many pre-frail older adults who may have benefited greatly from metformin. This requires further examination, as it is known that frail and pre-frail individuals often have issues with glucose homeostasis [74, 75], as well as impaired flu vaccine responders [44].

Metformin treatment induced trending increases in cTfh following vaccination. cTfh cells are essential for providing B cell help for efficient antibody responses following vaccination. Others have shown reduced cTfh responses with aging [42, 43]. More specifically, older adults had higher baseline activation of cTfh prior to vaccination and reduced upregulation of activated cTfh in response to vaccination compared to younger adults [43]. While we did not observe changes in activated cTfh with metformin treatment, it led to increased upregulation of total cTfh following vaccination. Interestingly, others have shown no alterations in total cTfh in older adults following flu vaccination [76]. It is possible that increased cTfh could lead to improved cTfh activation or other vaccination responses that were not evident in our study timeline. Additionally, it has been shown that activated cTfh from aged individuals have an elevated inflammatory signature with increased IL-2-STAT5 and TNF-NF-κB signaling compared to young individuals [77]. Since it is known that metformin can reduce inflammation through various mechanisms [22, 23], it is possible metformin treatment may improve the transcriptomic profile of cTfh. Future studies should focus on determining inflammatory profile and overall functionality of vaccine-induced cTfh to determine if metformin may improve flu vaccination responses.

Metformin treatment decreased CD57 expression, an exhaustion marker that identifies T cells with low proliferative capacity that are likely terminally differentiated [55, 56, 78], on CD4 T cells over the 20 weeks of treatment. This indicates that metformin treatment may have the potential to reduce T cell exhaustion and influence overall T cell function in healthy nondiabetic older adults. Metformin has previously been shown to reduce the replicative senescence and cell death associated with prolonged in vitro culturing [79], as well as act as a senostatic drug by reprogramming the senescence associated secretory phenotype (SASP) [80]. Our data further highlights the potential of metformin to have senolytic/SASP inhibition properties to reduce replicative senescence and/or age-associated declines in circulating CD4 T cells. Additionally, Mannick et al. previously showed that treatment with the mTOR inhibitor RAD001 decreases expression of the T cell exhaustion marker PD-1 in CD4 and CD8 T cells of older adults [34]. Although, we did not find differences in PD-1 expression with metformin on CD4 T cells, the change in CD57 expression shows that metformin reduces some aspects of T cell exhaustion. Conversely, we observed a trending increase in PD-1 expression on CD8 T cells, indicating that metformin may have differential effects on these different T cell populations. More research is necessary to delineate these different effects in CD4 and CD8 T cells and their impact overall immune responses with aging. It’s possible that metformin may have differential effects on various immune populations, and it will be important to determine the effects of metformin on these individual cell populations with future research.

Metformin can decrease various markers of inflammation in diabetics and other disease settings, however the effect of metformin on systemic inflammation in healthy, non-prediabetic and nondiabetic older adults has not been studied in depth. Metformin has previously been shown to decrease circulating levels of CRP, IL-6, and TNF-α in patients with type 2 diabetes [81], however this finding was not replicated in our subjects who were nondiabetic and nonprediabetic. It is likely that since our subjects were quite healthy with very limited co-morbidities and frailty levels that metformin did not have a measurable effect on circulating inflammatory levels. It is possible that less robust older adults may have benefits from metformin on systemic inflammation that were not apparent in our cohort. Interestingly, it was previously shown that CD4 T cells from older adults had elevated IL-6 and IL-17 production following polyclonal stimulation, and that in vitro treatment with metformin treatment could reduce this pro-inflammatory profile [20]. In vivo metformin treatment in our cohort of older adults did not replicate those findings in terms of circulating IL-6 and IL-17 levels. It’s likely that in vitro treatment does not replicate the overall aged microenvironment and may not replicate in vivo metformin concentrations in the tissues and plasma. Further, while metformin may impact cytokine secretion from CD4 T cells upon activation, it is not clear if this would cause measurable differences in circulating cytokines as multiple cell types contribute to the overall circulating inflammatory profile. Thus, it is possible that metformin treatment may have effects on CD4 T cell cytokine profile in certain contexts that was not observable in our study. More research is necessary to determine if the metformin benefits on CD4 T cells observed in vitro translate to meaningful differences in in vivo CD4 T cell function.

Metformin is known to impact IGF-1 concentrations, but its effect is dependent on the population as well as the duration of treatment [64]. We did not observe any differences in circulating IGF-1, however we observed increased IGFBP-2 with metformin treatment compared with placebo. Metformin is known to stimulate IGFBP-2 in diabetic patients [66], thus our findings agree in nondiabetic older adults. We also saw that metformin increased concentrations of GDF-15 and osteopontin, factors known to be influenced by metformin use [67, 68]. GDF-15 has been shown to mediate the effects of metformin on body weight and energy balance [67]. Interestingly, GDF15 is being investigated as a possible aging biomarker, and proteomic studies have shown that GDF15 is one of the biomarkers most strongly correlated with chronological age [82-84]. In older adults, increased concentrations of GDF-15 are associated with frailty, disability, reduced physical function, and worsened metabolism [82, 85, 86]. Similarly, others have also shown that osteopontin and GDF-15 are associated with age and frailty, while osteopontin, GDF-15, and MMP-2 are predicative of adverse post-surgery outcomes [87]. Despite the increase in GDF-15 and osteopontin in the metformin subjects, we saw no changes to frailty scores in either treatment group. While we didn’t see differences in weight loss between groups, since GDF-15 plays a major role in metformin-induce weight loss, it’s possible that increased GDF-15 may be temporary and not sustained as it is with frailty. The effect of metformin on GDF-15 and the resulting impact on energy balance and healthy aging requires additional research. In contrast to the association with frailty in some populations [87], osteopontin has been shown to attenuate aging-associated deficits in hematopoietic stem cells, specifically reducing the myeloid skewing seen with aging and restoring the proliferative capacity [88]. We did not evaluate hematopoietic stem cells in our study; thus, it is unclear if increased osteopontin could have a positive impact in older adults. Finally, we examined MMP2 and MMP9, extracellular matrix metalloproteinases that have been associated with long lived individuals [89]. While MMP2 was not affected, metformin treatment led to increased circulating MMP9. In totality, these findings demonstrate that short-term metformin use in healthy, nondiabetic/nonprediabetic older adults may replicate some promising findings, as seen in diabetic patients and have some potential pro-longevity effects. However, additional studies should examine the potential impact of metformin-induced increases in GDF-15 and osteopontin on physical function and frailty among nondiabetic older adults.

Although the majority of the research regarding metformin use in aged populations has been established in older diabetics, our pilot study highlights the potential ability of metformin treatment to combat age-related declines in vaccine responses and immune exhaustion. Ultimately, these results and continued research on the varying benefits of metformin will allow for a more comprehensive understanding on how to safely improve vaccination responses in older adults. Despite heterogeneity in aged immune responses, we showed that metformin treatment positively affected some immune parameters such as increased cTfh cells and reduced CD4 T cell exhaustion. Metformin is already a candidate drug used to target many of the biological drivers of aging due to its pleotropic effects [22, 23]. Combined with this, our study suggests that metformin may have additional utility for improvements in immunological resilience and vaccination responses in nondiabetic and nonprediabetic older adults. It is also possible that metformin may have more prominent immune benefits in older adults who are prediabetic, prefrail, frail, or have other comorbidities that were excluded from this pilot trial. Therefore, future research should explore the effects of metformin on vaccination responses and overall immune resilience in a more heterogeneous population of older adults.

## Conclusions

Since vaccination is one of the most critical forms of protection against infectious disease illness and mortality, ways to improve vaccination responses in at-risk older adults is critical. Here, we show that repurposing metformin, an already FDA approved drug with an established safety profile, may be a novel way to improve age-related immune declines. While metformin treatment did not improve antibody responses in this pilot trial, participants on metformin had increased cTfh post flu vaccination, a key component of flu vaccine responses known to be deficient in older adults. Furthermore, metformin treatment reduced CD4 T cell exhaustion marker CD57 expression. Thus, metformin treatment was safe and provided some enhancement of immunological resilience in nondiabetic/nonprediabetic older adults. Further investigation with a larger sample size and more heterogeneous aged population is needed to fully determine if metformin can increase flu vaccine induced protection in this vulnerable population.

## Methods

### Study design

Our double-blinded, placebo-controlled study randomized patients to either metformin or placebo treatment for approximately 20 weeks. An experimental timeline is detailed in Fig. 1A. The study protocol was approved by the Institutional Review Board at the University of Connecticut Health Center and registered at ClinicalTrials.gov (NCT03996538). All study participants provided written informed consent to participate in the study. Subjects were screened for eligibility, and if eligible, were randomized to metformin (final dose 1500 mg extended release (ER)/day) or placebo. To limit gastrointestinal issues per current metformin label recommendations, participants started with 1 tablet a day for week 1 (500 mg metformin ER/day or placebo), then 2 tablets a day for week 2 (1000 mg metformin ER or placebo), and finally 3 tablets a day for week 3 until the completion of the study (1500 mg metformin ER or placebo). Participants were vaccinated with Fluzone high-dose trivalent flu vaccine (Sanofi Pasteur Inc., Swiftwater, PA) after ~ 10 weeks of treatment. Blood was drawn prior to treatment (week -10, Baseline), immediately prior to vaccination (week 0), and 1-, 5-, and 10-weeks post vaccination. Adverse events were monitored by staff inquiry at all study visits and biweekly telephone calls throughout the study. Additional safety blood chemistry labs were performed following approximately 15 weeks of treatment.

### Study participants

Nineteen healthy older adults (> 65 years old) were enrolled in the study. All subjects underwent rigorous screening prior to randomization, including obtaining complete medical history and medication history, as well as general clinical blood labs. Exclusion criteria included the following: any unstable medical conditions or severe co-morbidities (severe COPD, severe congestive heart failure, advanced neurological disorders, etc.), contraindications for metformin (severe renal or liver impairment), contraindication for flu vaccine (history of Guillain–Barre syndrome post vaccination or allergic to component of vaccine), immunosuppressive disorders, immunosuppressive medications, and active cancer or history of metastatic cancer. Importantly, participants were excluded if they were prediabetic or diabetic (HbA1c ≥ 5.7%) to avoid any confounding impact of metformin on diabetes status. Participants were randomized to either metformin or placebo medication. Fifteen subjects (*n* = 8 metformin, *n* = 7 placebo, Table 1) completed the study on treatment medication with > 95% adherence (via dispensation/collection counts) and were used for our planned per-protocol data analysis. Treatment was well-tolerated with no serious adverse events and limited adverse events overall (Sup Table 1). Four subjects (2 metformin and 2 placebo) were not included in data analysis due to discontinuing treatment during the study, however no pattern of reason for discontinuation was observed (Sup Table 2). No differences were observed in basic demographics and characteristics between groups at baseline or following approximately 20 weeks of treatment (Table 1).

The small sample size is a major limitation of the study. While the intention was to recruit for both the 2019–2020 and 2020–2021 flu season, the COVID-19 pandemic impeded recruitment for the 2020–2021 flu season. Additionally, we had a higher screen failure rate than anticipated primarily due to undiagnosed prediabetes. Indeed, 23% of participants screened for the study were ineligible due to elevated HbA1c (≥ 5.7%), while renal insufficiency only accounted for a small percentage of screen failures. These findings highlight the high prevalence of undiagnosed prediabetes among older adults. Similarly, others have previously shown that the prevalence of undiagnosed diabetes increases in older adults to approximately 14.4% [90]. We observed similarly high rates of undiagnosed prediabetes in older adults. This may suggest the need for more regular preventative care in healthy older adults to better recognize and diagnose prediabetes early.

### Vaccination

Following approximately 10 weeks of treatment, blood was drawn and then Fluzone high dose inactivated trivalent flu vaccine (Fluzone High-dose, Sanofi Pasteur Inc., Swiftwater, PA) was administered intramuscularly to each subject.

### Blood processing

Blood was processed immediately for PBMCs, serum, and plasma at each study visit. PBMCs were isolated using the standard Ficoll method [91, 92] and cryopreserved in Human AB serum with 10% DMSO and maintained in liquid nitrogen. EDTA-treated whole blood was centrifuged for plasma isolation, while whole blood without additive was allowed to clot at room temperature for at least 20 min prior to centrifugation. Plasma and serum were immediately stored at -80C.

### Basic blood parameters and blood chemistries

Clinical blood parameters and chemistries, including CBC with differential, HbA1c, AST/ALT, Creatinine, Sodium, Chloride, Carbon Dioxide, Albumin, Glucose, and eGFR were analyzed by a CLIA certified clinical laboratory (Quest Diagnostics, Wallingford, CT) at screening, baseline, and after 15 weeks of treatment.

### Serum antibody titers

Hemagglutination inhibition assays (HIA) were performed using standard methods to determine antibody titers [49, 93]. Influenza subtypes used for HIA matched the strains used in the seasonal flu vaccine and were as follows: A/Brisbane/02/2018 (H1N1)pdm09-like virus; an A/Kansas/14/2017 (H3N2)-like virus; a B/Colorado/06/2017-like virus (B/Victoria lineage). All analyses were done in batches following completion of the study. Geometric Mean Titers (GMT) were calculated and antibody responses are expressed as fold change relative to pre-vaccination GMT.

### Cell-mediated immune response measures

Cell mediated immune responses were assessed as previously described [40, 49, 53, 94]. Briefly, thawed PBMCs were stimulated with sucrose-gradient purified, live influenza virus (A/Victoria/3/75 or B/Lee/40) (Charles River Laboratories, Wilmington, MA) at a multiplicity of infection of 2 in AIM V media (Thermo Fisher Scientific, Waltham, MA) and incubated at 37 °C/5% CO2 for 20 h. Supernatants and lysates were collected and stored at − 80 °C until assay measurement. Concentrations of IFNγ and IL-10 were measured in supernatants by multiplexed bead ELISA (Biorad Laboratories, Hercules, CA) and reported as pg/mL. PBMC lysates were analyzed for GrB activity as previously described [40, 49, 53, 94]. GrB activity was normalized to total protein content via BCA assay (Thermo Fisher Scientific, Waltham, MA). Inducible GrB (iGrB) was calculated by dividing the normalized stimulated GrB levels to normalized unstimulated GrB to account for basal GrB [40].

### Flow cytometry

PBMCs were analyzed via flow cytometry. For analysis of T cell subsets, cells were stained with CD3, CD4, CD57, ICOS, PD-1, CD8, CXCR5, CD45RA, and CCR7. For analysis of B cell subsets, cells were stained with CD19, CD24, CD27, CD38, and IgD. Samples were analyzed on the ZE5 Cell Analyzer (Bio-Rad Laboratories, Hercules, CA), and data were analyzed with FlowJo software (BD Biosciences, Woburn, MA). Full information on the antibodies used for flow cytometry experiments are in Table 2.

**Table 2:** Antibodies used for flow cytometry immune cell phenotyping in human peripheral blood mononuclear cells (PBMCs)

Antibody	Fluorochrome	Clone	Manufacturer
CD3	BV 605	OKT3	Biolegend
CD4	BUV 496	SK3	BD Biosciences
CD8	BUV 737	SK1	BD Biosciences
CD57	PE-Dazzle 594	HNK-1	Biolegend
ICOS	BV 421	C398.4A	Biolegend
PD-1	PE	EH12.2H7	Biolegend
CXCR5	BV 510	RF8B2	BD Biosciences
CD197 (CCR7)	APC-Fire 750	GO43H7	Biolegend
CD45RA	PE-Cy7	HI100	Biolegend
CD19	BUV 661	H1B19	BD Biosciences
CD24	BV 510	ML5	Biolegend
CD27	BV 605	M-T271	Biolegend
CD38	BV 421	H1T2	Biolegend
IgD	PerCP-Cy5.5	IA6-2	Biolegend
Carboxylic acid, succinimidylester (live/dead)	Alexa Fluor 350		Life Technologies

### Circulating cytokines, chemokines, and other factors

Cytokines, chemokines, and other factors were measured in EDTA plasma or serum at baseline and following ~ 20 weeks of treatment. Plasma IGF-1 was measured via ELISA (R&D Systems, dg100b, Minneapolis, MN). Plasma IL-6, IL-8, IL-10, CRP, TNFa, TNFRI, TNFRII, IGF-1, IGFBP-2, GDF-15, and Osteopontin were measured via multiplex (R&D Systems, LXSAHM, FCSTM18, Minneapolis, MN). Serum MMP-2 and MMP-9 were measured via multiplex (R&D Systems, FCSTM07, Minneapolis, MN).

### Statistical analysis

Data were graphed in Prism Graphpad (Graphpad Software, San Diego, CA) and results were analyzed by two-way repeated measure (RM) ANOVA with Geisser-Greenhouse correction and pairwise Šídák’s posthoc corrections or unpaired t-test as indicated. Data are presented as mean ± SEM. Significance was set at *p* ≤ 0.05.

## Supplementary Information

**Additional file 1: Supplemental Table 1.** Adverse effects by treatment group. Adverse events were monitored by staff inquiry at all study visits and biweekly telephone calls throughout the study. Safety blood chemistry labs were performed following approximately 15 weeks of treatment. Adverse events were recorded and broadly classified.

**Additional file 2: Supplemental Table 2.** Subjects who discontinued treatment during the study. Four participants chose to discontinue treatment during the study and the reasons given were recorded. No clear pattern was noted for metformin discontinuation.

**Additional file 3: Supplemental Figure 1.** MFI of PDI and ICOS expression on circulating Tfh. Peripheral blood mononuclear cells (PBMCs) were analyzed for circulating T follicular helper cells. Mean fluorescence intensity of the activation markers ICOS and PD1 was analyzed. Fold change was calculated from **A-B**) pre-vaccination and **C-D**) pre-treatment levels to determine the effect of metformin on cTfh activation prior to and following vaccination, respectively. Statistical significance was calculated by two-way repeated measures ANOVA with Šídák’s posthoc corrections and significance set at *p*<0.05.

**Additional file 4: Supplemental Figure 2.** Raw frequency of cTfh and ASC. Peripheral blood mononuclear cells (PBMCs) were analyzed for circulating T follicular helper cells (cTfh), activated ICOS+PD1+ cTfh, and Antibody Secreting Cells (ASCs). Frequency of **A**) cTfh, **B**) activated cTfh, and **C**) ASCs. **D-F**) Fold change was calculated from pre-treatment to determine the effect of metformin on these populations prior to vaccination. Statistical significance was calculated by two-way repeated measures ANOVA with Šídák’s posthoc corrections and significance set at *p*<0.05.

**Additional file 5: Supplemental Figure 3.** T and B Cell phenotypes. Peripheral blood mononuclear cells (PBMCs) were gated on CD4 and CD8 T cells and analyzed for naïve (CCR7+CD45RA+), central memory (CCR7+CD45RA-), effector memory (CCR7-CD45RA-), and terminally differentiated effector memory cells re-expressing CD45RA (TEMRA, CCR7-CD45RA+). Fold change was calculated from pre-treatment to determine the effect of metformin on these populations prior to vaccination. No differences between placebo and metformin group were observed in **A-D**) CD4 or **E-H**) CD8 T cells for these populations. PBMCs were also analyzed for B cell populations via gating on CD19+ cells and then phenotyping for **I**) naïve (IgD+CD27-),** J**) non-switched memory B cells (IgD+CD27+), **K**) switched memory B cells (IgD-CD27+), and **L**) double negative B cells (IgD-CD27-). Fold change was calculated from pre-treatment to determine the effect of metformin on populations prior to vaccination. Statistical significance was calculated by two-way repeated measures ANOVA with Šídák’s posthoc corrections and significance set at *p*<0.05.

**Additional file 6: Supplemental Figure 4.** Frequency of CD57+ CD4 and CD8 T cells. Frequency of CD57+ **A**) CD4 T cells and **B**) CD8 T cells prior to and following 20 weeks of treatment with either placebo or metformin was analyzed and fold change in frequency of CD57+ cells were calculated. Statistical significance was calculated by t-test and significance set at *p*<0.05.

**Additional file 7: Supplemental Figure 5.** Flow cytometric gating strategy for T cell populations. Peripheral blood mononuclear cells (PBMCs) were stained with antibodies as indicated in Table 2. Samples were analyzed on the ZE5 Cell Analyzer (Bio-Rad Laboratories, Hercules, CA), and data were analyzed with FlowJo software (BD Biosciences, Woburn, MA). Samples were first gated on lymphocytes (FSC-A x SCA-A), singularity (FSC-A x FSC-H), and identified as live with Carboxylic acid, succinimidyl ester (live/dead) staining prior to gating strategy illustrated.

**Additional file 8: Supplemental Figure 6.** Flow cytometric gating strategy for B cell populations. Peripheral blood mononuclear cells (PBMCs) were stained with antibodies as indicated in Table 2. Samples were analyzed on the ZE5 Cell Analyzer (Bio-Rad Laboratories, Hercules, CA), and data were analyzed with FlowJo software (BD Biosciences, Woburn, MA). Samples were first gated on lymphocytes (FSC-A x SCA-A), singularity (FSC-A x FSC-H), and identified as live with Carboxylic acid, succinimidyl ester (live/dead) staining prior to gating strategy illustrated.

## Data Availability

The datasets used and/or analysed during the current study are available from the corresponding author on reasonable request.
