# Supplementary material for: The effect of metformin on influenza vaccine responses in nondiabetic older adults: a pilot trial
Source: Immun Ageing. 2023 May 2;20:18. doi: 10.1186/s12979-023-00343-x (PMC10152024; doi:10.1186/s12979-023-00343-x)
Supplement: Supplementary file 1 — Additional file 1: Supplemental Table 1. Adverse effects by treatment group. Adverse events were monitored by staff inquiry at all study visits and biweekly telephone calls throughout the study. Safety blood chemistry labs were performed following approximately 15 weeks of treatment. Adverse events were recorded and broadly classified. [file 12979_2023_343_MOESM1_ESM.pdf]

Supp Table 1

	Supplementary Table 1. Adverse Events by Treatment Group	
**Side Effect**	Placebo (n)	Metformin (n)
Headache	0	2
Fatigue	0	1
Gastrointestinal Discomfort	2	1
Anemia	0	1
