# Supplementary material for: The effect of metformin on influenza vaccine responses in nondiabetic older adults: a pilot trial
Source: Immun Ageing. 2023 May 2;20:18. doi: 10.1186/s12979-023-00343-x (PMC10152024; doi:10.1186/s12979-023-00343-x)
Supplement: Supplementary file 2 — Additional file 2: Supplemental Table 2. Subjects who discontinued treatment during the study. Four participants chose to discontinue treatment during the study and the reasons given were recorded. No clear pattern was noted for metformin discontinuation. [file 12979_2023_343_MOESM2_ESM.pdf]

Supp Table 2

	Supplementary Table 2. Reasons for Treatment Discontinuation	
**Reason**	Placebo (n)	Metformin (n)
Difficulty swallowing pills	0	1
Fatigue and myalgia	0	1
Gastrointestinal Discomfort	2	0
