# Supplementary figures and images for: The effect of metformin on influenza vaccine responses in nondiabetic older adults: a pilot trial
Source: Immun Ageing. 2023 May 2;20:18. doi: 10.1186/s12979-023-00343-x (PMC10152024; doi:10.1186/s12979-023-00343-x)

Supplemental Figure 1

**A**

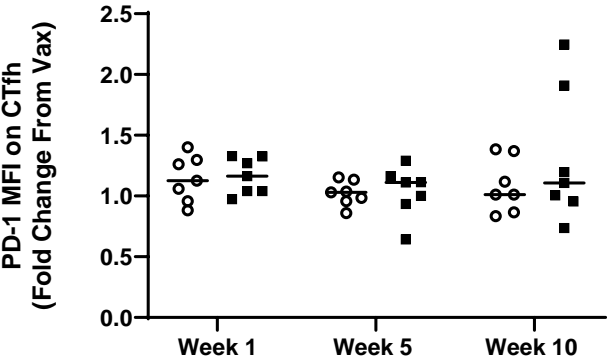

**B**

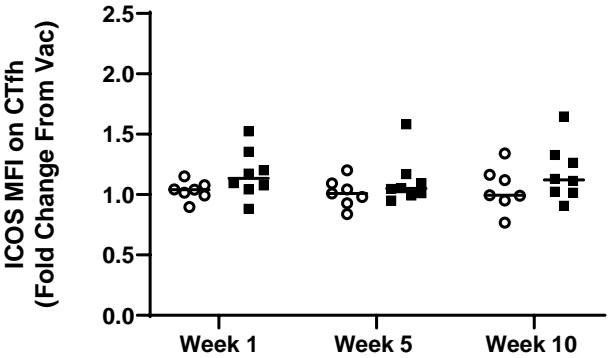

○ Placebo  
■ Metformin

**C**

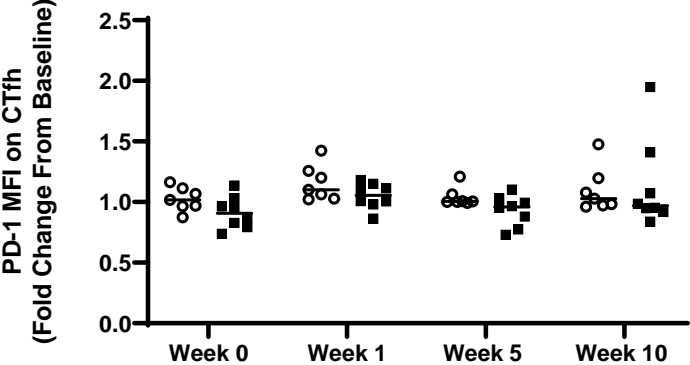

**D**

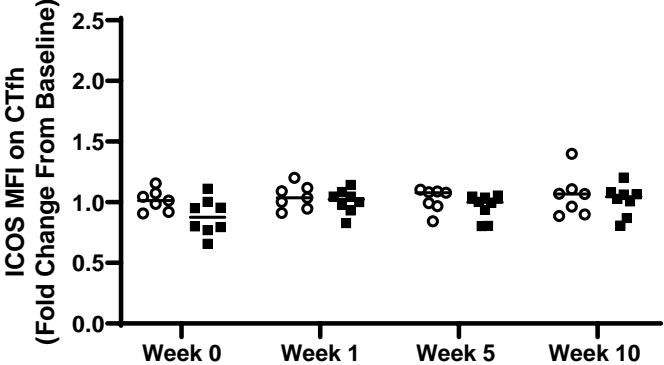

Supplement: Supplementary file 3 — Additional file 3: Supplemental Figure 1. MFI of PDI and ICOS expression on circulating Tfh. Peripheral blood mononuclear cells (PBMCs) were analyzed for circulating T follicular helper cells. Mean fluorescence intensity of the activation markers ICOS and PD1 was analyzed. Fold change was calculated from A-B) pre-vaccination and C-D) pre-treatment levels to determine the effect of metformin on cTfh activation prior to and following vaccination, respectively. Statistical significance was calculated by two-way repeated measures ANOVA with Šídák’s posthoc corrections and significance set at p<0.05. [file 12979_2023_343_MOESM3_ESM.pdf]

Supplemental Figure 2

**A**

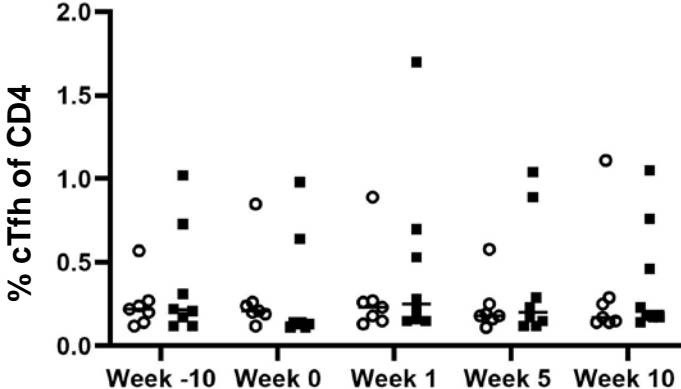

**B**

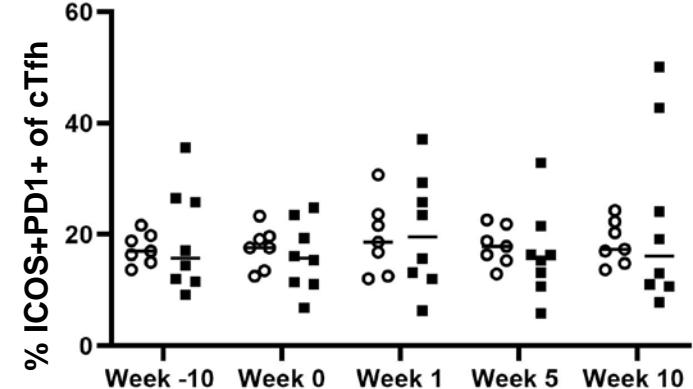

**C**

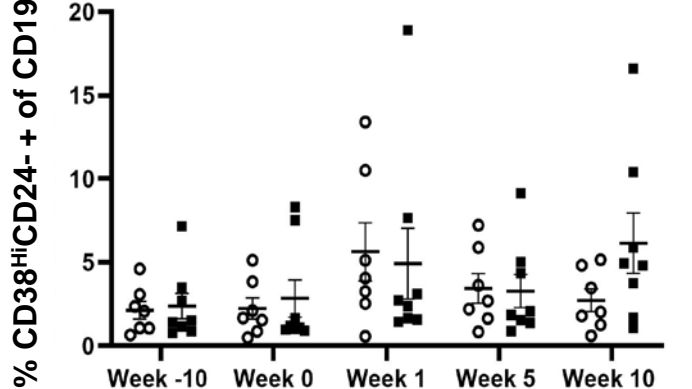

**D**

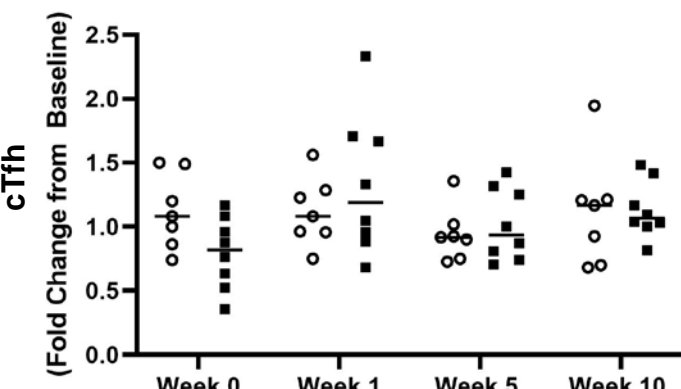

**E**

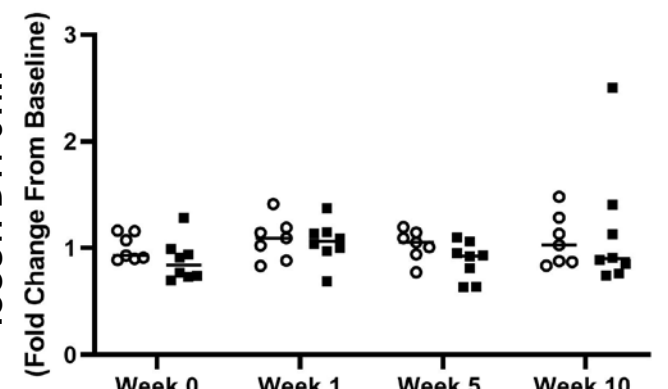

**D**

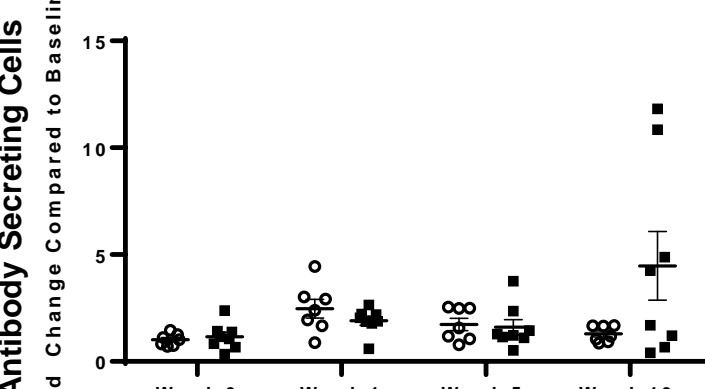

○ Placebo  
■ Metformin

Supplement: Supplementary file 4 — Additional file 4: Supplemental Figure 2. Raw frequency of cTfh and ASC. Peripheral blood mononuclear cells (PBMCs) were analyzed for circulating T follicular helper cells (cTfh), activated ICOS+PD1+ cTfh, and Antibody Secreting Cells (ASCs). Frequency of A) cTfh, B) activated cTfh, and C) ASCs. D-F) Fold change was calculated from pre-treatment to determine the effect of metformin on these populations prior to vaccination. Statistical significance was calculated by two-way repeated measures ANOVA with Šídák’s posthoc corrections and significance set at p<0.05. [file 12979_2023_343_MOESM4_ESM.pdf]

Supplemental Figure 3

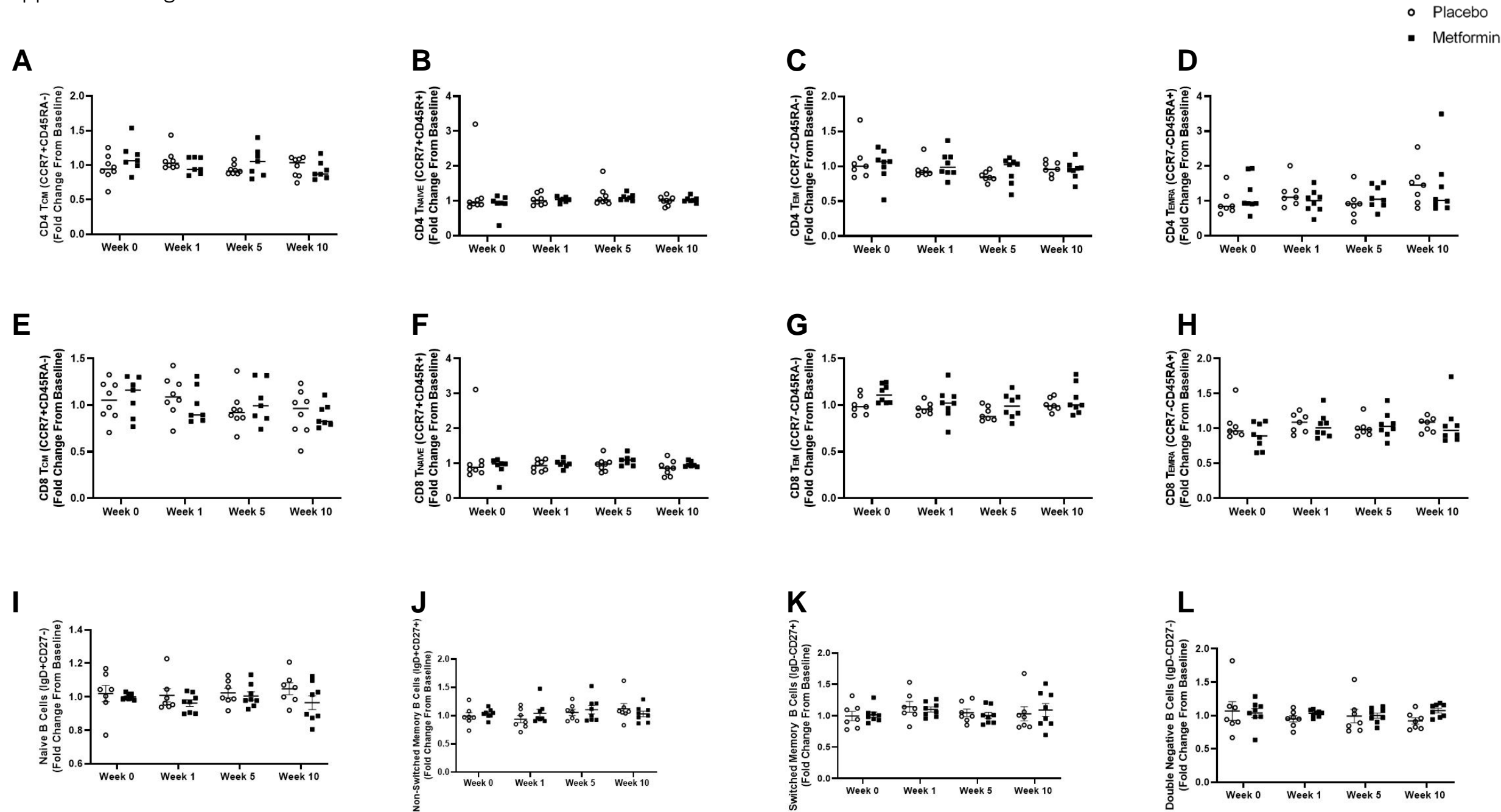

Supplement: Supplementary file 5 — Additional file 5: Supplemental Figure 3. T and B Cell phenotypes. Peripheral blood mononuclear cells (PBMCs) were gated on CD4 and CD8 T cells and analyzed for naïve (CCR7+CD45RA+), central memory (CCR7+CD45RA-), effector memory (CCR7-CD45RA-), and terminally differentiated effector memory cells re-expressing CD45RA (TEMRA, CCR7-CD45RA+). Fold change was calculated from pre-treatment to determine the effect of metformin on these populations prior to vaccination. No differences between placebo and metformin group were observed in A-D) CD4 or E-H) CD8 T cells for these populations. PBMCs were also analyzed for B cell populations via gating on CD19+ cells and then phenotyping for I) naïve (IgD+CD27-), J) non-switched memory B cells (IgD+CD27+), K) switched memory B cells (IgD-CD27+), and L) double negative B cells (IgD-CD27-). Fold change was calculated from pre-treatment to determine the effect of metformin on populations prior to vaccination. Statistical significance was calculated by two-way repeated measures ANOVA with Šídák’s posthoc corrections and significance set at p<0.05. [file 12979_2023_343_MOESM5_ESM.pdf]

# A

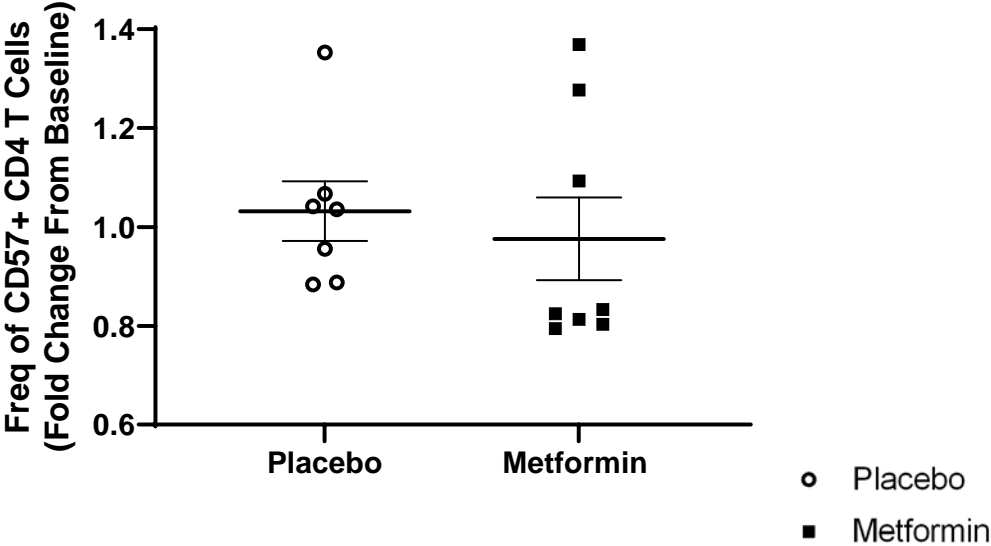

# B

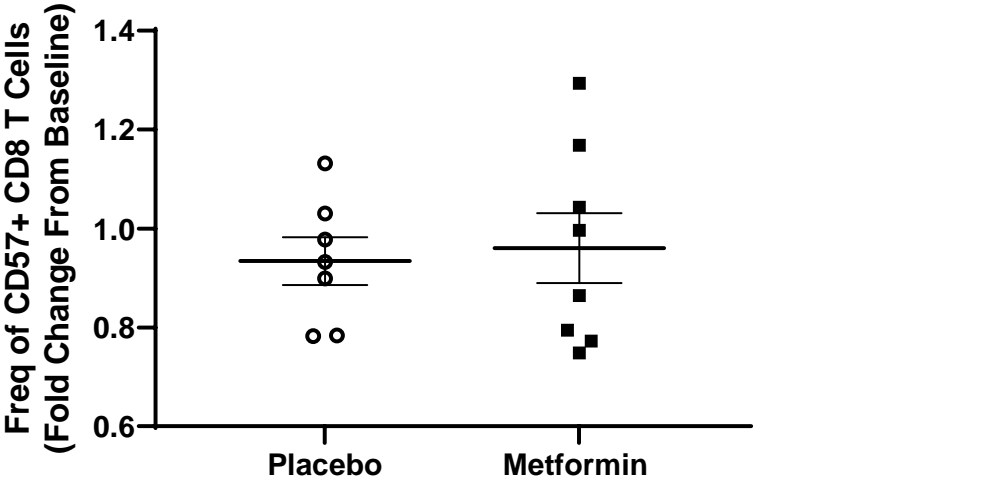

Supplement: Supplementary file 6 — Additional file 6: Supplemental Figure 4. Frequency of CD57+ CD4 and CD8 T cells. Frequency of CD57+ A) CD4 T cells and B) CD8 T cells prior to and following 20 weeks of treatment with either placebo or metformin was analyzed and fold change in frequency of CD57+ cells were calculated. Statistical significance was calculated by t-test and significance set at p<0.05. [file 12979_2023_343_MOESM6_ESM.pdf]

Supplemental Figure 5

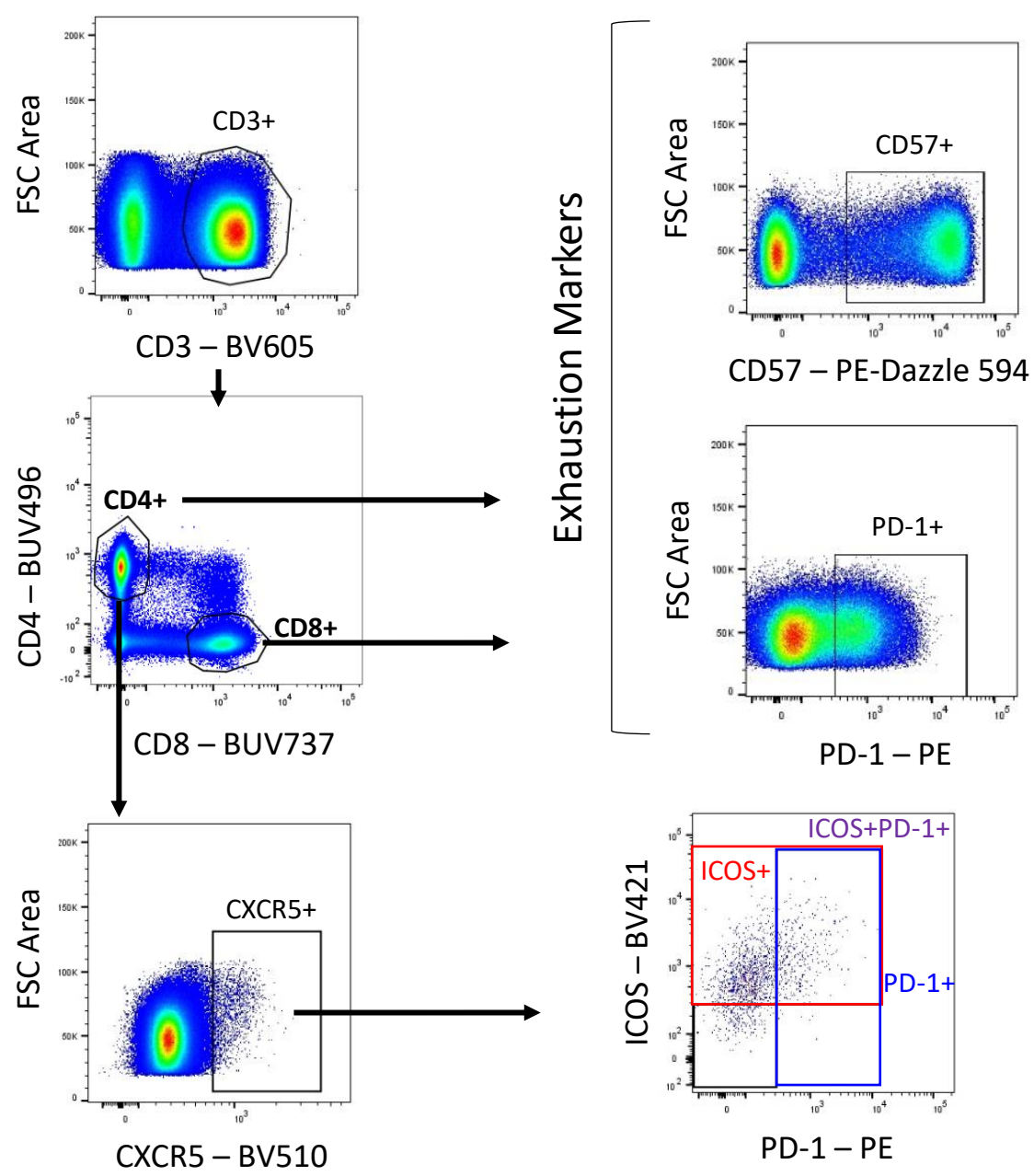

Supplement: Supplementary file 7 — Additional file 7: Supplemental Figure 5. Flow cytometric gating strategy for T cell populations. Peripheral blood mononuclear cells (PBMCs) were stained with antibodies as indicated in Table 2. Samples were analyzed on the ZE5 Cell Analyzer (Bio-Rad Laboratories, Hercules, CA), and data were analyzed with FlowJo software (BD Biosciences, Woburn, MA). Samples were first gated on lymphocytes (FSC-A x SCA-A), singularity (FSC-A x FSC-H), and identified as live with Carboxylic acid, succinimidyl ester (live/dead) staining prior to gating strategy illustrated. [file 12979_2023_343_MOESM7_ESM.pdf]
