# Supplementary material for: The effect of metformin on influenza vaccine responses in nondiabetic older adults: a pilot trial
Source: Immun Ageing. 2023 May 2;20:18. doi: 10.1186/s12979-023-00343-x (PMC10152024; doi:10.1186/s12979-023-00343-x)
Supplement: Supplementary file 8 — Additional file 8: Supplemental Figure 6. Flow cytometric gating strategy for B cell populations. Peripheral blood mononuclear cells (PBMCs) were stained with antibodies as indicated in Table 2. Samples were analyzed on the ZE5 Cell Analyzer (Bio-Rad Laboratories, Hercules, CA), and data were analyzed with FlowJo software (BD Biosciences, Woburn, MA). Samples were first gated on lymphocytes (FSC-A x SCA-A), singularity (FSC-A x FSC-H), and identified as live with Carboxylic acid, succinimidyl ester (live/dead) staining prior to gating strategy illustrated. [file 12979_2023_343_MOESM8_ESM.pdf]

Supplemental Figure 6

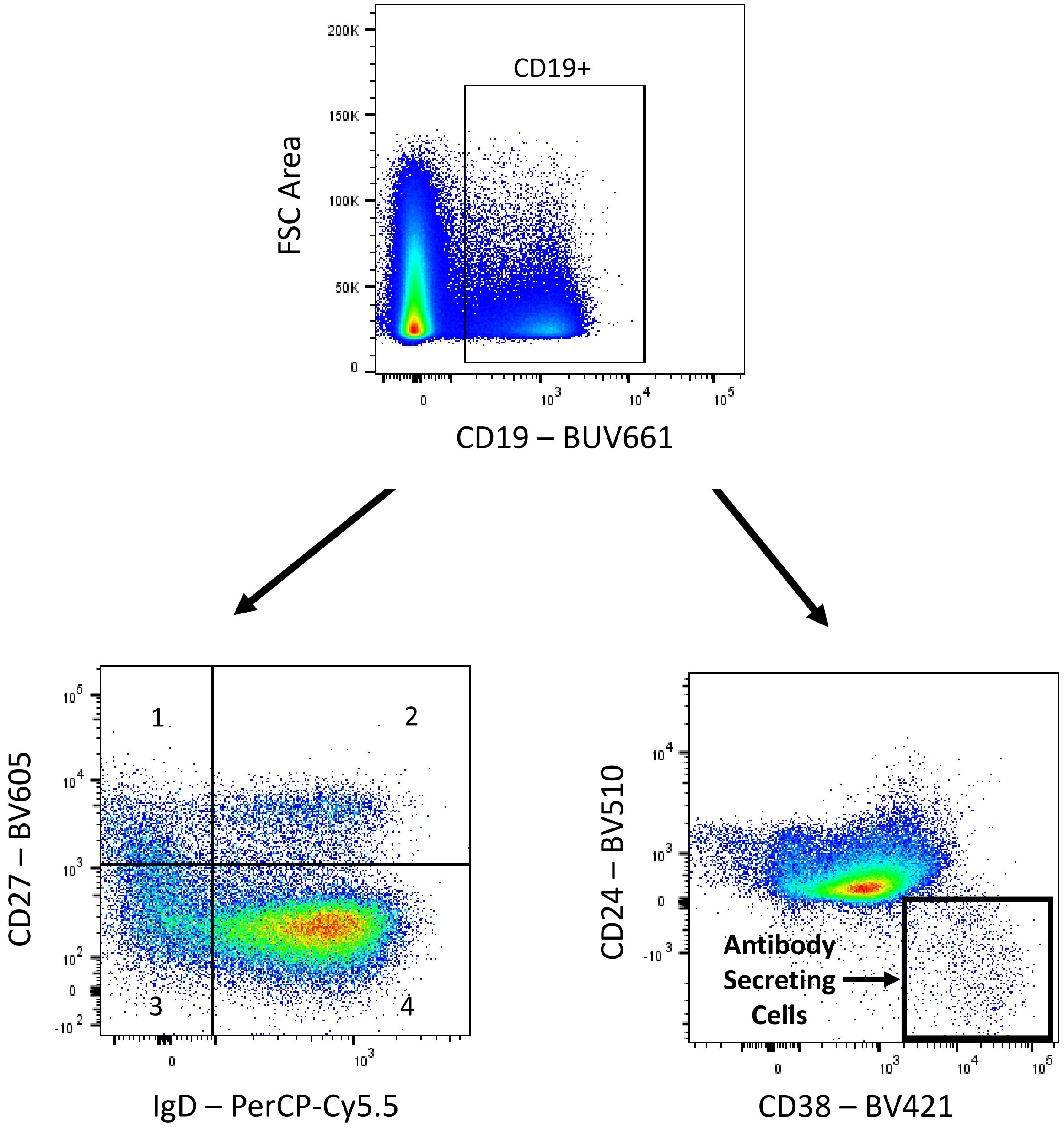

1 – IgD+CD27+ Switched Memory B Cells
2 – IgD-CD27+ Naïve B Cells
3 – IgD-CD27- Double Negative B Cells
4 – IgD+CD27- Un-switched Memory B Cells
